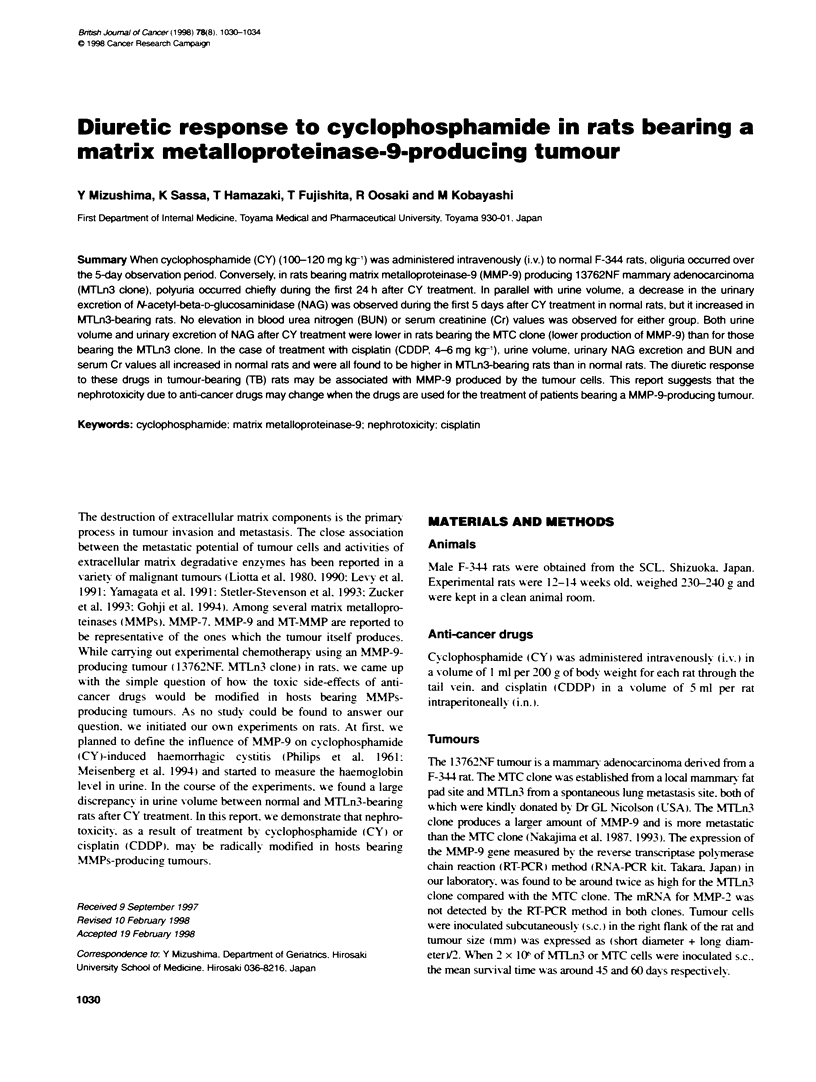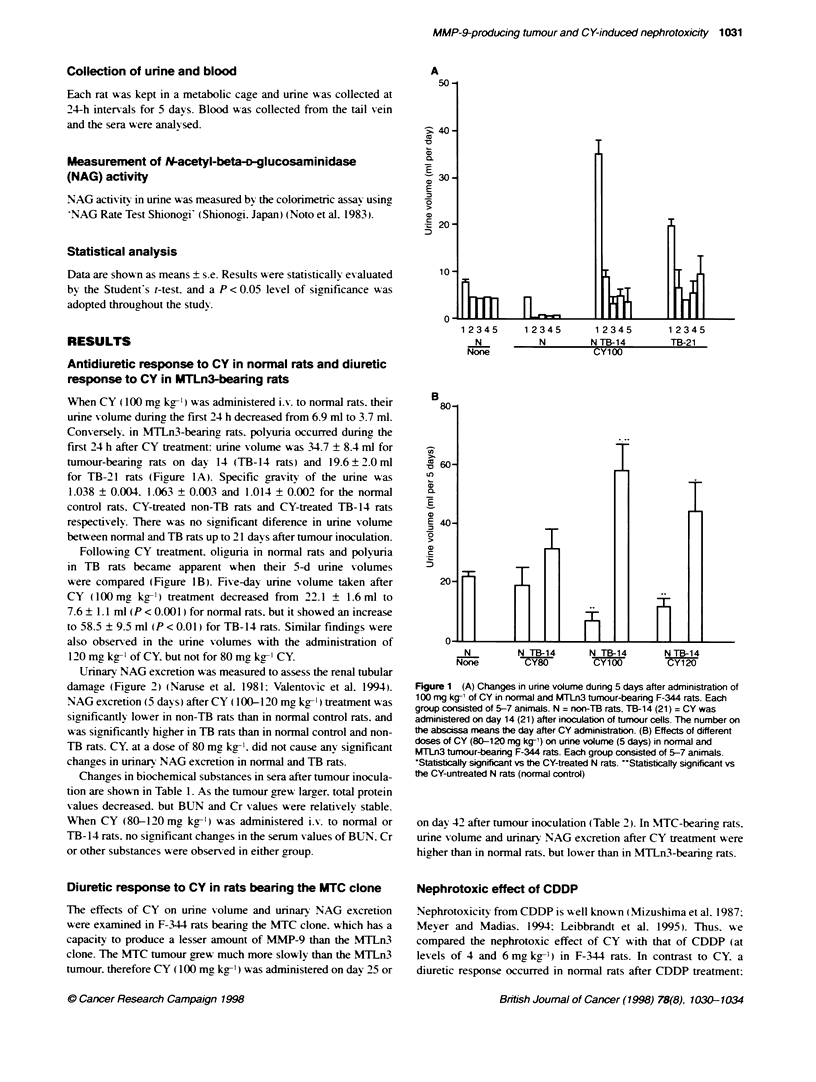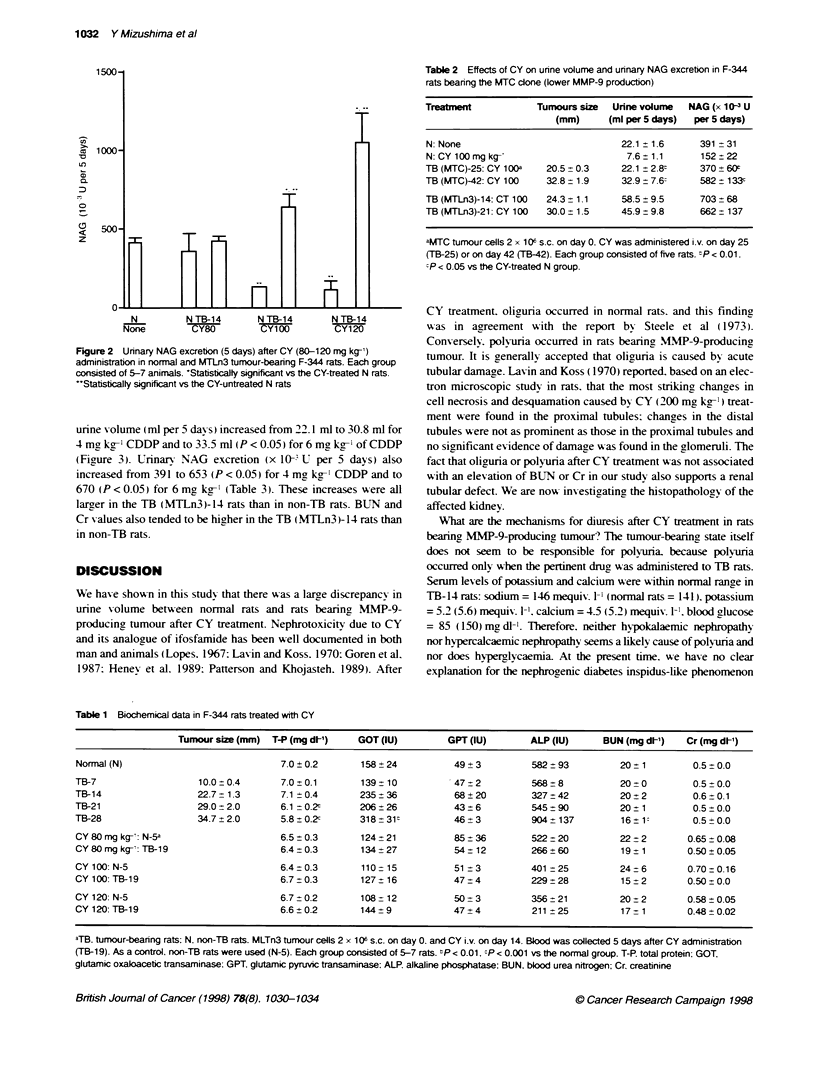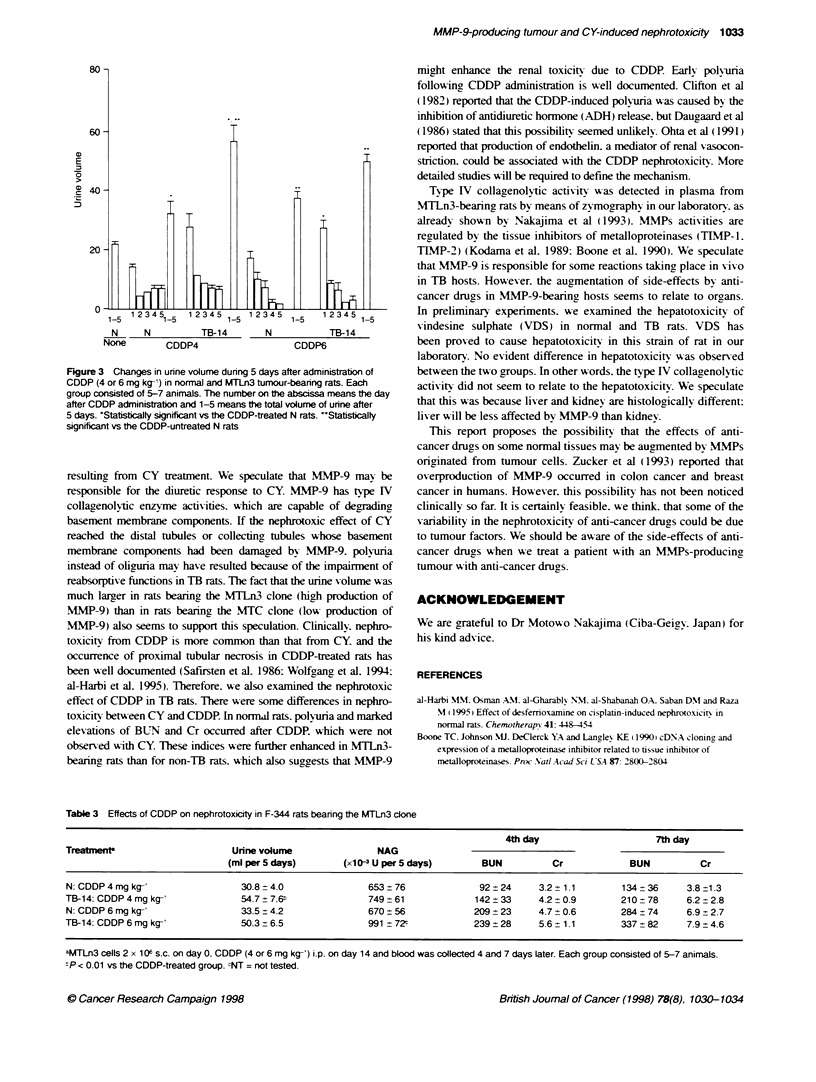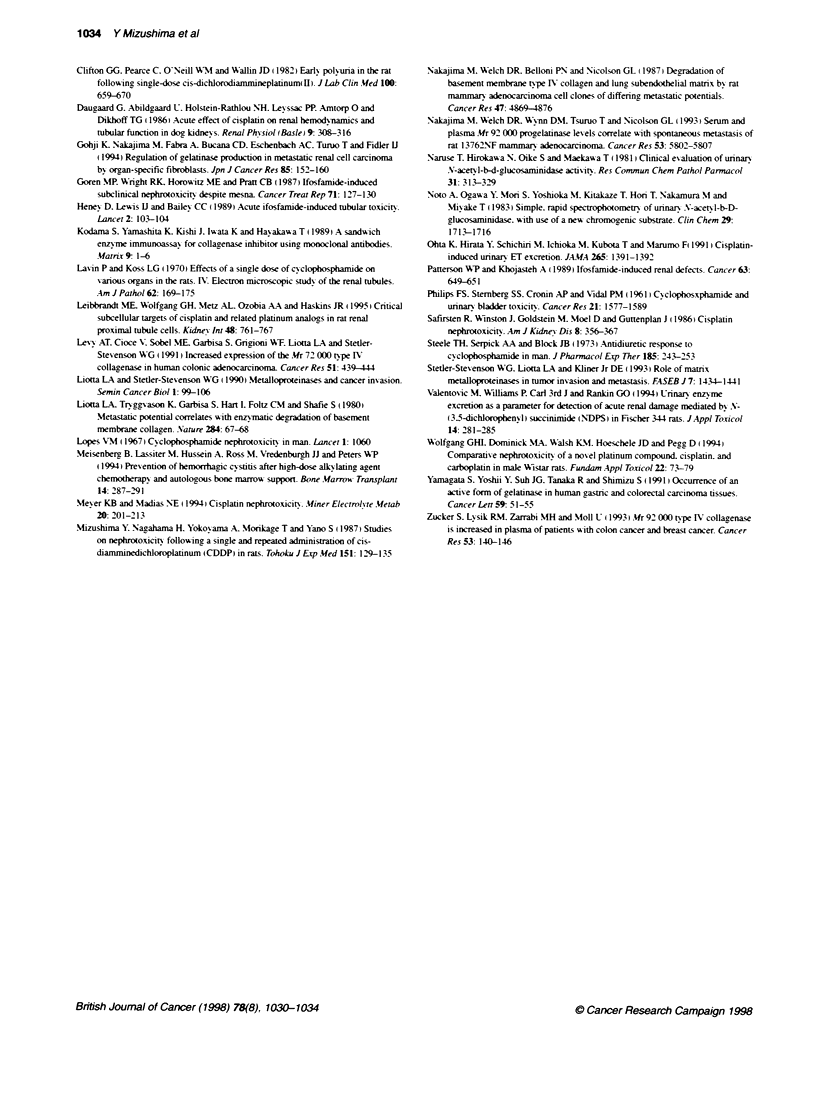# Diuretic response to cyclophosphamide in rats bearing a matrix metalloproteinase-9-producing tumour.

**DOI:** 10.1038/bjc.1998.623

**Published:** 1998-10

**Authors:** Y. Mizushima, K. Sassa, T. Hamazaki, T. Fujishita, R. Oosaki, M. Kobayashi

**Affiliations:** First Department of Internal Medicine, Toyama Medical and Pharmaceutical University, Japan.

## Abstract

When cyclophosphamide (CY) (100-120 mg kg(-1)) was administered intravenously (i.v.) to normal F-344 rats, oliguria occurred over the 5-day observation period. Conversely, in rats bearing matrix metalloproteinase-9 (MMP-9) producing 13762NF mammary adenocarcinoma (MTLn3 clone), polyuria occurred chiefly during the first 24 h after CY treatment. In parallel with urine volume, a decrease in the urinary excretion of N-acetyl-beta-D-glucosaminidase (NAG) was observed during the first 5 days after CY treatment in normal rats, but it increased in MTLn3-bearing rats. No elevation in blood urea nitrogen (BUN) or serum creatinine (Cr) values was observed for either group. Both urine volume and urinary excretion of NAG after CY treatment were lower in rats bearing the MTC clone (lower production of MMP-9) than for those bearing the MTLn3 clone. In the case of treatment with cisplatin (CDDP, 4-6 mg kg(-1)), urine volume, urinary NAG excretion and BUN and serum Cr values all increased in normal rats and were all found to be higher in MTLn3-bearing rats than in normal rats. The diuretic response to these drugs in tumour-bearing (TB) rats may be associated with MMP-9 produced by the tumour cells. This report suggests that the nephrotoxicity due to anti-cancer drugs may change when the drugs are used for the treatment of patients bearing a MMP-9-producing tumour.


British Journal of Cancer (1998) 78(8), 1030–1034
© 1998 Cancer Research Campaign

### Diuretic response to cyclophosphamide in rats bearing a matrix metalloproteinase-9-producing tumour

**Y Mizushima, K Sassa, T Hamazaki, T Fujishita, R Oosaki and M Kobayashi**

First Department of Internal Medicine, Toyama Medical and Pharmaceutical University, Toyama 930-01, Japan

**Summary** When cyclophosphamide (CY) (100–120 mg $kg^{-1}$) was administered intravenously (i.v.) to normal F-344 rats, oliguria occurred over the 5-day observation period. Conversely, in rats bearing matrix metalloproteinase-9 (MMP-9) producing 13762NF mammary adenocarcinoma (MTLn3 clone), polyuria occurred chiefly during the first 24 h after CY treatment. In parallel with urine volume, a decrease in the urinary excretion of *N*-acetyl-beta-D-glucosaminidase (NAG) was observed during the first 5 days after CY treatment in normal rats, but it increased in MTLn3-bearing rats. No elevation in blood urea nitrogen (BUN) or serum creatinine (Cr) values was observed for either group. Both urine volume and urinary excretion of NAG after CY treatment were lower in rats bearing the MTC clone (lower production of MMP-9) than for those bearing the MTLn3 clone. In the case of treatment with cisplatin (CDDP, 4–6 mg $kg^{-1}$), urine volume, urinary NAG excretion and BUN and serum Cr values all increased in normal rats and were all found to be higher in MTLn3-bearing rats than in normal rats. The diuretic response to these drugs in tumour-bearing (TB) rats may be associated with MMP-9 produced by the tumour cells. This report suggests that the nephrotoxicity due to anti-cancer drugs may change when the drugs are used for the treatment of patients bearing a MMP-9-producing tumour.

**Keywords:** cyclophosphamide; matrix metalloproteinase-9; nephrotoxicity; cisplatin

The destruction of extracellular matrix components is the primary process in tumour invasion and metastasis. The close association between the metastatic potential of tumour cells and activities of extracellular matrix degradative enzymes has been reported in a variety of malignant tumours (Liotta et al, 1980, 1990; Levy et al, 1991; Yamagata et al, 1991; Stetler-Stevenson et al, 1993; Zucker et al, 1993; Gohji et al, 1994). Among several matrix metalloproteinases (MMPs), MMP-7, MMP-9 and MT-MMP are reported to be representative of the ones which the tumour itself produces. While carrying out experimental chemotherapy using an MMP-9-producing tumour (13762NF, MTLn3 clone) in rats, we came up with the simple question of how the toxic side-effects of anti-cancer drugs would be modified in hosts bearing MMPs-producing tumours. As no study could be found to answer our question, we initiated our own experiments on rats. At first, we planned to define the influence of MMP-9 on cyclophosphamide (CY)-induced haemorrhagic cystitis (Philips et al, 1961; Meisenberg et al, 1994) and started to measure the haemoglobin level in urine. In the course of the experiments, we found a large discrepancy in urine volume between normal and MTLn3-bearing rats after CY treatment. In this report, we demonstrate that nephrotoxicity, as a result of treatment by cyclophosphamide (CY) or cisplatin (CDDP), may be radically modified in hosts bearing MMPs-producing tumours.

*Received 9 September 1997*
*Revised 10 February 1998*
*Accepted 19 February 1998*

*Correspondence to:* Y Mizushima, Department of Geriatrics, Hirosaki University School of Medicine, Hirosaki 036-8216, Japan

### MATERIALS AND METHODS

### Animals

Male F-344 rats were obtained from the SCL, Shizuoka, Japan. Experimental rats were 12–14 weeks old, weighed 230–240 g and were kept in a clean animal room.

### Anti-cancer drugs

Cyclophosphamide (CY) was administered intravenously (i.v.) in a volume of 1 ml per 200 g of body weight for each rat through the tail vein, and cisplatin (CDDP) in a volume of 5 ml per rat intraperitoneally (i.p.).

### Tumours

The 13762NF tumour is a mammary adenocarcinoma derived from a F-344 rat. The MTC clone was established from a local mammary fat pad site and MTLn3 from a spontaneous lung metastasis site, both of which were kindly donated by Dr GL Nicolson (USA). The MTLn3 clone produces a larger amount of MMP-9 and is more metastatic than the MTC clone (Nakajima et al, 1987, 1993). The expression of the MMP-9 gene measured by the reverse transcriptase polymerase chain reaction (RT-PCR) method (RNA-PCR kit, Takara, Japan) in our laboratory, was found to be around twice as high for the MTLn3 clone compared with the MTC clone. The mRNA for MMP-2 was not detected by the RT-PCR method in both clones. Tumour cells were inoculated subcutaneously (s.c.) in the right flank of the rat and tumour size (mm) was expressed as (short diameter + long diameter)/2. When $2 \times 10^6$ of MTLn3 or MTC cells were inoculated s.c., the mean survival time was around 45 and 60 days respectively.

### Collection of urine and blood

Each rat was kept in a metabolic cage and urine was collected at 24-h intervals for 5 days. Blood was collected from the tail vein and the sera were analysed.

### Measurement of *N*-acetyl-beta-D-glucosaminidase (NAG) activity

NAG activity in urine was measured by the colorimetric assay using 'NAG Rate Test Shionogi' (Shionogi, Japan) (Noto et al. 1983).

### Statistical analysis

Data are shown as means ± s.e. Results were statistically evaluated by the Student's *t*-test, and a $P < 0.05$ level of significance was adopted throughout the study.

### RESULTS

### Antidiuretic response to CY in normal rats and diuretic response to CY in MTLn3-bearing rats

When CY (100 mg $kg^{-1}$) was administered i.v. to normal rats, their urine volume during the first 24 h decreased from 6.9 ml to 3.7 ml. Conversely, in MTLn3-bearing rats, polyuria occurred during the first 24 h after CY treatment: urine volume was 34.7 ± 8.4 ml for tumour-bearing rats on day 14 (TB-14 rats) and 19.6 ± 2.0 ml for TB-21 rats (Figure 1A). Specific gravity of the urine was 1.038 ± 0.004, 1.063 ± 0.003 and 1.014 ± 0.002 for the normal control rats, CY-treated non-TB rats and CY-treated TB-14 rats respectively. There was no significant diference in urine volume between normal and TB rats up to 21 days after tumour inoculation.

Following CY treatment, oliguria in normal rats and polyuria in TB rats became apparent when their 5-d urine volumes were compared (Figure 1B). Five-day urine volume taken after CY (100 mg $kg^{-1}$) treatment decreased from 22.1 ± 1.6 ml to 7.6 ± 1.1 ml ($P < 0.001$) for normal rats, but it showed an increase to 58.5 ± 9.5 ml ($P < 0.01$) for TB-14 rats. Similar findings were also observed in the urine volumes with the administration of 120 mg $kg^{-1}$ of CY, but not for 80 mg $kg^{-1}$ CY.

Urinary NAG excretion was measured to assess the renal tubular damage (Figure 2) (Naruse et al. 1981; Valentovic et al. 1994). NAG excretion (5 days) after CY (100–120 mg $kg^{-1}$) treatment was significantly lower in non-TB rats than in normal control rats, and was significantly higher in TB rats than in normal control and non-TB rats. CY, at a dose of 80 mg $kg^{-1}$, did not cause any significant changes in urinary NAG excretion in normal and TB rats.

Changes in biochemical substances in sera after tumour inoculation are shown in Table 1. As the tumour grew larger, total protein values decreased, but BUN and Cr values were relatively stable. When CY (80–120 mg $kg^{-1}$) was administered i.v. to normal or TB-14 rats, no significant changes in the serum values of BUN, Cr or other substances were observed in either group.

**Figure 1** (A) Changes in urine volume during 5 days after administration of 100 mg $kg^{-1}$ of CY in normal and MTLn3 tumour-bearing F-344 rats. Each group consisted of 5–7 animals. N = non-TB rats, TB-14 (21) = CY was administered on day 14 (21) after inoculation of tumour cells. The number on the abscissa means the day after CY administration. (B) Effects of different doses of CY (80–120 mg $kg^{-1}$) on urine volume (5 days) in normal and MTLn3 tumour-bearing F-344 rats. Each group consisted of 5–7 animals. *Statistically significant vs the CY-treated N rats. **Statistically significant vs the CY-untreated N rats (normal control)

### Diuretic response to CY in rats bearing the MTC clone

The effects of CY on urine volume and urinary NAG excretion were examined in F-344 rats bearing the MTC clone, which has a capacity to produce a lesser amount of MMP-9 than the MTLn3 clone. The MTC tumour grew much more slowly than the MTLn3 tumour, therefore CY (100 mg $kg^{-1}$) was administered on day 25 or on day 42 after tumour inoculation (Table 2). In MTC-bearing rats, urine volume and urinary NAG excretion after CY treatment were higher than in normal rats, but lower than in MTLn3-bearing rats.

### Nephrotoxic effect of CDDP

Nephrotoxicity from CDDP is well known (Mizushima et al. 1987; Meyer and Madias, 1994; Leibbrandt et al. 1995). Thus, we compared the nephrotoxic effect of CY with that of CDDP (at levels of 4 and 6 mg $kg^{-1}$) in F-344 rats. In contrast to CY, a diuretic response occurred in normal rats after CDDP treatment;

**Figure 2** Urinary NAG excretion (5 days) after CY (80–120 mg $kg^{-1}$) administration in normal and MTLn3 tumour-bearing F-344 rats. Each group consisted of 5–7 animals. *Statistically significant vs the CY-treated N rats. **Statistically significant vs the CY-untreated N rats urine volume (ml per 5 days) increased from 22.1 ml to 30.8 ml for 4 mg $kg^{-1}$ CDDP and to 33.5 ml ($P < 0.05$) for 6 mg $kg^{-1}$ of CDDP (Figure 3). Urinary NAG excretion ($\times 10^{-3}$ U per 5 days) also increased from 391 to 653 ($P < 0.05$) for 4 mg $kg^{-1}$ CDDP and to 670 ($P < 0.05$) for 6 mg $kg^{-1}$ (Table 3). These increases were all larger in the TB (MTLn3)-14 rats than in non-TB rats. BUN and Cr values also tended to be higher in the TB (MTLn3)-14 rats than in non-TB rats.

### DISCUSSION

We have shown in this study that there was a large discrepancy in urine volume between normal rats and rats bearing MMP-9-producing tumour after CY treatment. Nephrotoxicity due to CY and its analogue of ifosfamide has been well documented in both man and animals (Lopes, 1967; Lavin and Koss, 1970; Goren et al, 1987; Heney et al, 1989; Patterson and Khojasteh, 1989). After

**Table 2** Effects of CY on urine volume and urinary NAG excretion in F-344 rats bearing the MTC clone (lower MMP-9 production)

| Treatment | Tumours size (mm) | Urine volume (ml per 5 days) | NAG ($\times 10^{-3}$ U per 5 days) |
|---|---|---|---|
| N: None | | 22.1 ± 1.6 | 391 ± 31 |
| N: CY 100 mg $kg^{-1}$ | | 7.6 ± 1.1 | 152 ± 22 |
| TB (MTC)-25: CY 100[a] | 20.5 ± 0.3 | 22.1 ± 2.8[b] | 370 ± 60[c] |
| TB (MTC)-42: CY 100 | 32.8 ± 1.9 | 32.9 ± 7.6[c] | 582 ± 133[c] |
| TB (MTLn3)-14: CT 100 | 24.3 ± 1.1 | 58.5 ± 9.5 | 703 ± 68 |
| TB (MTLn3)-21: CY 100 | 30.0 ± 1.5 | 45.9 ± 9.8 | 662 ± 137 |

[a]MTC tumour cells 2 × $10^6$ s.c. on day 0. CY was administered i.v. on day 25 (TB-25) or on day 42 (TB-42). Each group consisted of five rats. [b]$P < 0.01$. [c]$P < 0.05$ vs the CY-treated N group.

CY treatment, oliguria occurred in normal rats, and this finding was in agreement with the report by Steele et al (1973). Conversely, polyuria occurred in rats bearing MMP-9-producing tumour. It is generally accepted that oliguria is caused by acute tubular damage. Lavin and Koss (1970) reported, based on an electron microscopic study in rats, that the most striking changes in cell necrosis and desquamation caused by CY (200 mg $kg^{-1}$) treatment were found in the proximal tubules; changes in the distal tubules were not as prominent as those in the proximal tubules and no significant evidence of damage was found in the glomeruli. The fact that oliguria or polyuria after CY treatment was not associated with an elevation of BUN or Cr in our study also supports a renal tubular defect. We are now investigating the histopathology of the affected kidney.

What are the mechanisms for diuresis after CY treatment in rats bearing MMP-9-producing tumour? The tumour-bearing state itself does not seem to be responsible for polyuria, because polyuria occurred only when the pertinent drug was administered to TB rats. Serum levels of potassium and calcium were within normal range in TB-14 rats: sodium = 146 mequiv. $l^{-1}$ (normal rats = 141), potassium = 5.2 (5.6) mequiv. $l^{-1}$, calcium = 4.5 (5.2) mequiv. $l^{-1}$, blood glucose = 85 (150) mg $dl^{-1}$. Therefore, neither hypokalaemic nephropathy nor hypercalcaemic nephropathy seems a likely cause of polyuria and nor does hyperglycaemia. At the present time, we have no clear explanation for the nephrogenic diabetes inspidus-like phenomenon

**Table 1** Biochemical data in F-344 rats treated with CY

| | Tumour size (mm) | T-P (mg $dl^{-1}$) | GOT (IU) | GPT (IU) | ALP (IU) | BUN (mg $dl^{-1}$) | Cr (mg $dl^{-1}$) |
|---|---|---|---|---|---|---|---|
| Normal (N) | | 7.0 ± 0.2 | 158 ± 24 | 49 ± 3 | 582 ± 93 | 20 ± 1 | 0.5 ± 0.0 |
| TB-7 | 10.0 ± 0.4 | 7.0 ± 0.1 | 139 ± 10 | 47 ± 2 | 568 ± 8 | 20 ± 0 | 0.5 ± 0.0 |
| TB-14 | 22.7 ± 1.3 | 7.1 ± 0.4 | 235 ± 36 | 68 ± 20 | 327 ± 42 | 20 ± 2 | 0.6 ± 0.1 |
| TB-21 | 29.0 ± 2.0 | 6.1 ± 0.2[c] | 206 ± 26 | 43 ± 6 | 545 ± 90 | 20 ± 1 | 0.5 ± 0.0 |
| TB-28 | 34.7 ± 2.0 | 5.8 ± 0.2[c] | 318 ± 31[b] | 46 ± 3 | 904 ± 137 | 16 ± 1[c] | 0.5 ± 0.0 |
| CY 80 mg $kg^{-1}$: N-5[a] | | 6.5 ± 0.3 | 124 ± 21 | 85 ± 36 | 522 ± 20 | 22 ± 2 | 0.65 ± 0.08 |
| CY 80 mg $kg^{-1}$: TB-19 | | 6.4 ± 0.3 | 134 ± 27 | 54 ± 12 | 266 ± 60 | 19 ± 1 | 0.50 ± 0.05 |
| CY 100: N-5 | | 6.4 ± 0.3 | 110 ± 15 | 51 ± 3 | 401 ± 25 | 24 ± 6 | 0.70 ± 0.16 |
| CY 100: TB-19 | | 6.7 ± 0.3 | 127 ± 16 | 47 ± 4 | 229 ± 28 | 15 ± 2 | 0.50 ± 0.0 |
| CY 120: N-5 | | 6.7 ± 0.2 | 108 ± 12 | 50 ± 3 | 356 ± 21 | 20 ± 2 | 0.58 ± 0.05 |
| CY 120: TB-19 | | 6.6 ± 0.2 | 144 ± 9 | 47 ± 4 | 211 ± 25 | 17 ± 1 | 0.48 ± 0.02 |

[a]TB, tumour-bearing rats; N, non-TB rats. MLTn3 tumour cells 2 × $10^6$ s.c. on day 0, and CY i.v. on day 14. Blood was collected 5 days after CY administration (TB-19). As a control, non-TB rats were used (N-5). Each group consisted of 5–7 rats. [b]$P < 0.01$, [c]$P < 0.001$ vs the normal group. T-P, total protein; GOT, glutamic oxaloacetic transaminase; GPT, glutamic pyruvic transaminase; ALP, alkaline phosphatase; BUN, blood urea nitrogen; Cr, creatinine

© Cancer Research Campaign 1998

**Figure 3** Changes in urine volume during 5 days after administration of CDDP (4 or 6 mg kg⁻¹) in normal and MTLn3 tumour-bearing rats. Each group consisted of 5–7 animals. The number on the abscissa means the day after CDDP administration and 1–5 means the total volume of urine after 5 days. *Statistically significant vs the CDDP-treated N rats. **Statistically significant vs the CDDP-untreated N rats resulting from CY treatment. We speculate that MMP-9 may be responsible for the diuretic response to CY. MMP-9 has type IV collagenolytic enzyme activities, which are capable of degrading basement membrane components. If the nephrotoxic effect of CY reached the distal tubules or collecting tubules whose basement membrane components had been damaged by MMP-9, polyuria instead of oliguria may have resulted because of the impairment of reabsorptive functions in TB rats. The fact that the urine volume was much larger in rats bearing the MTLn3 clone (high production of MMP-9) than in rats bearing the MTC clone (low production of MMP-9) also seems to support this speculation. Clinically, nephrotoxicity from CDDP is more common than that from CY, and the occurrence of proximal tubular necrosis in CDDP-treated rats has been well documented (Safirsten et al, 1986; Wolfgang et al, 1994; al-Harbi et al, 1995). Therefore, we also examined the nephrotoxic effect of CDDP in TB rats. There were some differences in nephrotoxicity between CY and CDDP. In normal rats, polyuria and marked elevations of BUN and Cr occurred after CDDP, which were not observed with CY. These indices were further enhanced in MTLn3-bearing rats than for non-TB rats, which also suggests that MMP-9 might enhance the renal toxicity due to CDDP. Early polyuria following CDDP administration is well documented. Clifton et al (1982) reported that the CDDP-induced polyuria was caused by the inhibition of antidiuretic hormone (ADH) release, but Daugaard et al (1986) stated that this possibility seemed unlikely. Ohta et al (1991) reported that production of endothelin, a mediator of renal vasoconstriction, could be associated with the CDDP nephrotoxicity. More detailed studies will be required to define the mechanism.

Type IV collagenolytic activity was detected in plasma from MTLn3-bearing rats by means of zymography in our laboratory, as already shown by Nakajima et al (1993). MMPs activities are regulated by the tissue inhibitors of metalloproteinases (TIMP-1, TIMP-2) (Kodama et al, 1989; Boone et al, 1990). We speculate that MMP-9 is responsible for some reactions taking place in vivo in TB hosts. However, the augmentation of side-effects by anti-cancer drugs in MMP-9-bearing hosts seems to relate to organs. In preliminary experiments, we examined the hepatotoxicity of vindesine sulphate (VDS) in normal and TB rats. VDS has been proved to cause hepatotoxicity in this strain of rat in our laboratory. No evident difference in hepatotoxicity was observed between the two groups. In other words, the type IV collagenolytic activity did not seem to relate to the hepatotoxicity. We speculate that this was because liver and kidney are histologically different; liver will be less affected by MMP-9 than kidney.

This report proposes the possibility that the effects of anti-cancer drugs on some normal tissues may be augmented by MMPs originated from tumour cells. Zucker et al (1993) reported that overproduction of MMP-9 occurred in colon cancer and breast cancer in humans. However, this possibility has not been noticed clinically so far. It is certainly feasible, we think, that some of the variability in the nephrotoxicity of anti-cancer drugs could be due to tumour factors. We should be aware of the side-effects of anti-cancer drugs when we treat a patient with an MMPs-producing tumour with anti-cancer drugs.

### ACKNOWLEDGEMENT

We are grateful to Dr Motowo Nakajima (Ciba-Geigy, Japan) for his kind advice.

### REFERENCES

al-Harbi MM, Osman AM, al-Gharably NM, al-Shabanah OA, Saban DM and Raza M (1995) Effect of desferrioxamine on cisplatin-induced nephrotoxicity in normal rats. *Chemotherapy* **41**: 448–454

Boone TC, Johnson MJ, DeClerck YA and Langley KE (1990) cDNA cloning and expression of a metalloproteinase inhibitor related to tissue inhibitor of metalloproteinases. *Proc Natl Acad Sci USA* **87**: 2800–2804

**Table 3** Effects of CDDP on nephrotoxicity in F-344 rats bearing the MTLn3 clone

| Treatment[a] | Urine volume (ml per 5 days) | NAG ($\times 10^{-3}$ U per 5 days) | 4th day | | 7th day | |
|---|---|---|---|---|---|---|
| | | | BUN | Cr | BUN | Cr |
| N: CDDP 4 mg kg⁻¹ | 30.8 ± 4.0 | 653 ± 76 | 92 ± 24 | 3.2 ± 1.1 | 134 ± 36 | 3.8 ±1.3 |
| TB-14: CDDP 4 mg kg⁻¹ | 54.7 ± 7.6[b] | 749 ± 61 | 142 ± 33 | 4.2 ± 0.9 | 210 ± 78 | 6.2 ± 2.8 |
| N: CDDP 6 mg kg⁻¹ | 33.5 ± 4.2 | 670 ± 56 | 209 ± 23 | 4.7 ± 0.6 | 284 ± 74 | 6.9 ± 2.7 |
| TB-14: CDDP 6 mg kg⁻¹ | 50.3 ± 6.5 | 991 ± 72[b] | 239 ± 28 | 5.6 ± 1.1 | 337 ± 82 | 7.9 ± 4.6 |

[a]MTLn3 cells 2 × $10^6$ s.c. on day 0. CDDP (4 or 6 mg kg⁻¹) i.p. on day 14 and blood was collected 4 and 7 days later. Each group consisted of 5–7 animals. [b]$P < 0.01$ vs the CDDP-treated group. [c]NT = not tested.

© Cancer Research Campaign 1998 

Clifton GG, Pearce C, O'Neill WM and Wallin JD (1982) Early polyuria in the rat following single-dose cis-dichlorodiammineplatinum(II). *J Lab Clin Med* **100**: 659–670

Daugaard G, Abildgaard U, Holstein-Rathlou NH, Leyssac PP, Amtorp O and Dikhoff TG (1986) Acute effect of cisplatin on renal hemodynamics and tubular function in dog kidneys. *Renal Physiol (Basle)* **9**: 308–316

Gohji K, Nakajima M, Fabra A, Bucana CD, Eschenbach AC, Turuo T and Fidler IJ (1994) Regulation of gelatinase production in metastatic renal cell carcinoma by organ-specific fibroblasts. *Jpn J Cancer Res* **85**: 152–160

Goren MP, Wright RK, Horowitz ME and Pratt CB (1987) Ifosfamide-induced subclinical nephrotoxicity despite mesna. *Cancer Treat Rep* **71**: 127–130

Heney D, Lewis IJ and Bailey CC (1989) Acute ifosfamide-induced tubular toxicity. *Lancet* **2**: 103–104

Kodama S, Yamashita K, Kishi J, Iwata K and Hayakawa T (1989) A sandwich enzyme immunoassay for collagenase inhibitor using monoclonal antibodies. *Matrix* **9**: 1–6

Lavin P and Koss LG (1970) Effects of a single dose of cyclophosphamide on various organs in the rats. IV. Electron microscopic study of the renal tubules. *Am J Pathol* **62**: 169–175

Leibbrandt ME, Wolfgang GH, Metz AL, Ozobia AA and Haskins JR (1995) Critical subcellular targets of cisplatin and related platinum analogs in rat renal proximal tubule cells. *Kidney Int* **48**: 761–767

Levy AT, Cioce V, Sobel ME, Garbisa S, Grigioni WF, Liotta LA and Stetler-Stevenson WG (1991) Increased expression of the *Mr* 72 000 type IV collagenase in human colonic adenocarcinoma. *Cancer Res* **51**: 439–444

Liotta LA and Stetler-Stevenson WG (1990) Metalloproteinases and cancer invasion. *Semin Cancer Biol* **1**: 99–106

Liotta LA, Tryggvason K, Garbisa S, Hart I, Foltz CM and Shafie S (1980) Metastatic potential correlates with enzymatic degradation of basement membrane collagen. *Nature* **284**: 67–68

Lopes VM (1967) Cyclophosphamide nephrotoxicity in man. *Lancet* **1**: 1060

Meisenberg B, Lassiter M, Hussein A, Ross M, Vredenburgh JJ and Peters WP (1994) Prevention of hemorrhagic cystitis after high-dose alkylating agent chemotherapy and autologous bone marrow support. *Bone Marrow Transplant* **14**: 287–291

Meyer KB and Madias NE (1994) Cisplatin nephrotoxicity. *Miner Electrolyte Metab* **20**: 201–213

Mizushima Y, Nagahama H, Yokoyama A, Morikage T and Yano S (1987) Studies on nephrotoxicity following a single and repeated administration of cis-diamminedichloroplatinum (CDDP) in rats. *Tohoku J Exp Med* **151**: 129–135

Nakajima M, Welch DR, Belloni PN and Nicolson GL (1987) Degradation of basement membrane type IV collagen and lung subendothelial matrix by rat mammary adenocarcinoma cell clones of differing metastatic potentials. *Cancer Res* **47**: 4869–4876

Nakajima M, Welch DR, Wynn DM, Tsuruo T and Nicolson GL (1993) Serum and plasma *Mr* 92 000 progelatinase levels correlate with spontaneous metastasis of rat 13762NF mammary adenocarcinoma. *Cancer Res* **53**: 5802–5807

Naruse T, Hirokawa N, Oike S and Maekawa T (1981) Clinical evaluation of urinary N-acetyl-b-d-glucosaminidase activity. *Res Commun Chem Pathol Parmacol* **31**: 313–329

Noto A, Ogawa Y, Mori S, Yoshioka M, Kitakaze T, Hori T, Nakamura M and Miyake T (1983) Simple, rapid spectrophotometry of urinary N-acetyl-b-D-glucosaminidase, with use of a new chromogenic substrate. *Clin Chem* **29**: 1713–1716

Ohta K, Hirata Y, Schichiri M, Ichioka M, Kubota T and Marumo F (1991) Cisplatin-induced urinary ET excretion. *JAMA* **265**: 1391–1392

Patterson WP and Khojasteh A (1989) Ifosfamide-induced renal defects. *Cancer* **63**: 649–651

Philips FS, Sternberg SS, Cronin AP and Vidal PM (1961) Cyclophosxphamide and urinary bladder toxicity. *Cancer Res* **21**: 1577–1589

Safirsten R, Winston J, Goldstein M, Moel D and Guttenplan J (1986) Cisplatin nephrotoxicity. *Am J Kidney Dis* **8**: 356–367

Steele TH, Serpick AA and Block JB (1973) Antidiuretic response to cyclophosphamide in man. *J Pharmacol Exp Ther* **185**: 243–253

Stetler-Stevenson WG, Liotta LA and Kliner Jr DE (1993) Role of matrix metalloproteinases in tumor invasion and metastasis. *FASEB J* **7**: 1434–1441

Valentovic M, Williams P, Carl 3rd J and Rankin GO (1994) Urinary enzyme excretion as a parameter for detection of acute renal damage mediated by N-(3,5-dichlorophenyl) succinimide (NDPS) in Fischer 344 rats. *J Appl Toxicol* **14**: 281–285

Wolfgang GHI, Dominick MA, Walsh KM, Hoeschele JD and Pegg D (1994) Comparative nephrotoxicity of a novel platinum compound, cisplatin, and carboplatin in male Wistar rats. *Fundam Appl Toxicol* **22**: 73–79

Yamagata S, Yoshii Y, Suh JG, Tanaka R and Shimizu S (1991) Occurrence of an active form of gelatinase in human gastric and colorectal carcinoma tissues. *Cancer Lett* **59**: 51–55

Zucker S, Lysik RM, Zarrabi MH and Moll U (1993) *Mr* 92 000 type IV collagenase is increased in plasma of patients with colon cancer and breast cancer. *Cancer Res* **53**: 140–146

© Cancer Research Campaign 1998